# Distinct gut flora profile induced by postnatal trans-fat diet in gestationally bisphenol A-exposed rats

**DOI:** 10.1371/journal.pone.0306741

**Published:** 2024-07-09

**Authors:** Sarah Zulkifli, Noor Shafina Mohd Nor, Siti Hamimah Sheikh Abdul Kadir, Norashikin Mohd Ranai, Khalilah Abdul Khalil

**Affiliations:** 1 Institute for Pathology, Laboratory and Forensic Medicine (I-PPerForM), Faculty of Medicine, Universiti Teknologi MARA (UiTM) Sungai Buloh Campus, Selangor, Malaysia; 2 Department of Paediatrics, Faculty of Medicine, Universiti Teknologi MARA (UiTM) Sungai Buloh Campus, Selangor, Malaysia; 3 Institute of Medical Molecular Biotechnology, Faculty of Medicine, Universiti Teknologi MARA (UiTM) Sungai Buloh Campus, Selangor, Malaysia; 4 Department of Biochemistry and Molecular Medicine, Faculty of Medicine, Universiti Teknologi MARA (UiTM) Sungai Buloh Campus, Selangor, Malaysia; 5 Department of Biomolecular Sciences, Faculty of Applied Sciences, Universiti Teknologi MARA (UiTM) Shah Alam, Selangor, Malaysia; Nathan S Kline Institute, UNITED STATES

## Abstract

There has been much evidence showing the repercussions of prenatal bisphenol A (BPA) exposure with a postnatal high fat-diet (HFD) on offspring’s health. However, the information on how the interaction between these two variables affects the gut microbiome is rather limited. Hence, we investigated the impact of a postnatal trans fat diet (TFD) on the gut microbiome of offspring exposed to BPA during the prenatal period in an animal model. Pregnant rats were divided into 5 mg/kg/day BPA, vehicle Tween80 (P80) or control (CTL) drinking water until delivery (N = 6 per group). Then, weaned male pups were further subdivided into three normal diet (ND) groups (CTLND, P80ND, and BPAND) and three TFD groups (CTLTFD, P80TFD, and BPATFD) (n = 6 per group). 180–250 g of faecal samples were collected on days 50 and 100 to assess the composition of the offspring’s intestinal flora using next-generation sequencing. The alpha diversity indices of TFD offspring with and without BPA were markedly lower than their ND counterparts (p<0.001–p<0.05). The beta diversity, hierarchical cluster and network analyses of the offspring’s microbiome demonstrated that the microbiome species of the TFD group with and without BPA were distinctly different compared to the ND group. Consistently, TFD and ND offspring pairings exhibited a higher number of significantly different species (p<0.0001–p<0.05) compared to those exposed to prenatal BPA exposure and different life stages comparisons, as shown by the multivariate parametric analysis DESeq2. Predictive functional profiling of the offspring’s intestinal flora demonstrated altered expressions of genes involved in metabolic pathways. In summary, the gut flora composition of the rat offspring may be influenced by postnatal diet instead of prenatal exposure to BPA. Our data indicate the possibility of perturbed metabolic functions and epigenetic modifications, in offspring that consumed TFD, which may theoretically lead to metabolic diseases in middle or late adulthood. Further investigation is necessary to fully understand these implications.

## Introduction

Epoxy resins and polycarbonate plastic industries use bisphenol A (BPA) as the base material for their production. The main source of BPA exposure is through our dietary intake, such as canned food, bottled water, and food that is kept in plastic containers [1]. It was revealed that obese people living in semi-urban and industrial areas exhibited substantially higher urinary BPA levels than in urban and rural areas [1]. BPA has also been associated with various other diseases by affecting the structure and function of the systemic organs, such as the heart, pancreas, and brain [2]. The ubiquitous presence of BPA in the environment has been reported to be detrimental to health, especially for the developing foetus, even at lower doses. Studies have shown that BPA can cross the placenta in conjugated form (does not bind to oestrogen receptors) and is able to be detected at specific concentrations in the amniotic fluid, umbilical cord and foetal cord serum [2]. At the same time, this synthetic compound can be biotransformed and excreted in the urine. The immature foetal gut may increase the susceptibility of the foetus to the adverse effects of BPA where they perturb the normal programming of foetal organ development via epigenetic mechanisms, including DNA methylation.

Industrial trans fatty acid (TFA) is frequently used in processed food, where liquid vegetable oil is partially hydrogenated to increase its shelf life [3]. Higher consumption of trans-fat diets (TFDs) is more common among the disadvantaged socioeconomic groups [4, 5]. Therefore, this part of society also showed a higher rate of obesity and its co-morbidities [6–8]. The addition of TFA into manufactured food is banned in certain countries like the United States, South Africa, and Norway, while some make it compulsory to declare TFA on food labels; however, most of the third world countries which still do not have any mandatory legislation on TFA levels [9–11]. Unfortunately, TFA levels exceeding the limits (1.5 g/100g for solid foods and 0.75 g/100 ml for liquid foods) as regulated in the Malaysian Food Act 1983 can still be found in Malaysian processed food due to the lack of mandatory legislation on the TFA content declaration on food labels [12, 13]. Furthermore, industrial TFA intake has been associated with obesity development in children and adults, among other diseases [14, 15].

The intestinal flora is an ecosystem of microbes that live in the intestine, which has direct and indirect contributions to the physiological processes as well as forming a symbiotic relationship between the host and microbiota in the intestine. It is responsible for dietary fermentation in the colon, nutrient extraction, vitamin synthesis, metabolic release to the systemic tissues, immune system and intestinal epithelium maturation, nerve function and gastrointestinal hormone release modulation, as well as pathogen colonisation prevention [16]. These activities are controlled by the host genotype and environmental factors, which also affect the gut flora composition.

The intestinal flora plays an indispensable role in the onset and progression of obesity and other metabolic diseases via its composition and metabolites [17, 18]. Obese mouse models, either induced by exposure to BPA or by diet, displayed microbial imbalance in the gut [19–23]. Following either of these treatments, there was a decrease in the diversity of the intestinal flora and an increase in the relative abundance of “harmful" bacteria. In mouse offspring exposed to 50000 μg/kg BPA from the periconceptional period until lactation, higher abundances of *Methanobrevibacter* spp (related to increased adiposity [24]) and *Akkermansia* spp. (positively associated with insulin signalling and steroid synthesis pathways) and lower *Desulfovibrio* spp. abundance were observed [25]. Other than that, the mice that were directly exposed to BPA from week 4 to week 14 displayed a substantial decrease in the diversity of gut flora species, along with the populations of *Clostridia* and *Firmicutes*, whereas *Proteobacteria* and *Helicobacteraceae* growth were favoured, resembling high-fat diet (HFD) repercussions on the structure of the microbial community of their counterparts [20].

Meanwhile, in some cases, microbiome shift is the perpetrator of many disease processes including dysmetabolic morbidities, which act via bacterial translocation and elevated intestinal permeability [19, 26, 27]. For example, young mouse offspring that were exposed to BPA perinatally (from the second gestational trimester to day 21) had a lower abundance of *Bifidobacteria* than their vehicle counterparts at day 45, and some strains have been shown to resist inflammation [19]. Consistent with this, it was observed that faecal IgA secretion and antimicrobial activity, especially lysozyme activity, decreased in adulthood (day 170).

Therefore, this study was conducted to investigate the implications of gestational BPA exposure and postnatal TFD on the intestinal flora of rat offspring. This is because research has shown that early exposure to BPA and postnatal environmental factors such as a HFD and overfeeding can have an impact on the final phenotypic outcomes of offspring’s heart and metabolic health [28–30]. Regardless, the combined impacts of prenatal BPA insults and postnatal TFD on the gut microbial community structure are still an area to be discovered. This raises the question of whether the postnatal TFD could amplify the alterations in the intestinal flora programmed by prenatal BPA exposure, as previously investigated in the animal heart, adipose tissue and pancreas [28–31].

## Results

### Effects of prenatal BPA exposure and postnatal TFD on the alpha and beta diversity of adult rat offspring’s intestinal flora

**Fig 1:**
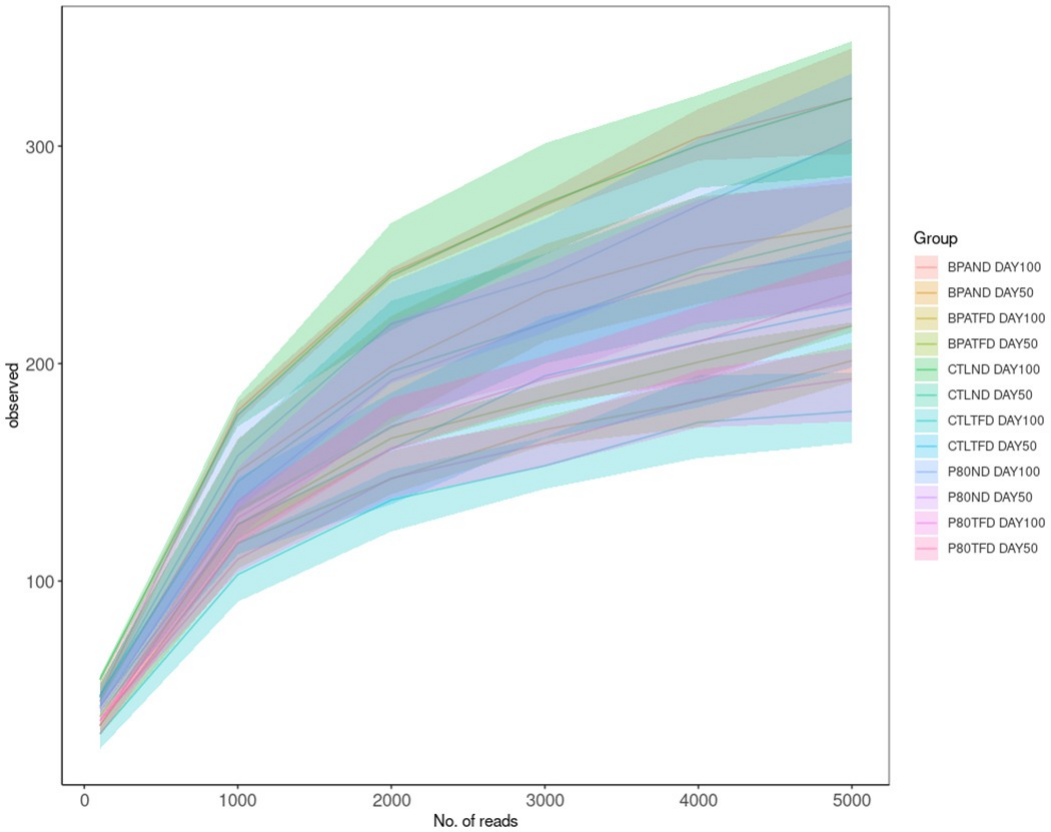
Rarefaction plot using the number of observed OTUs for each sample. The plot shows the proportion of genes that can be identified from the entire gene catalogue as the number of raw sequence reads increases.

The rarefaction curve was plotted to ascertain a proper sequencing depth allowing the observation of all microbial species in the rat offspring’s faecal samples. The number of operational taxonomic units (OTUs) initially increased and then stabilised as the number of sequence reads increased (Fig 1).

As shown in Fig 1, the faecal samples’ volume was sufficient to cover all the species for the microbial analysis.

**Fig 2:**
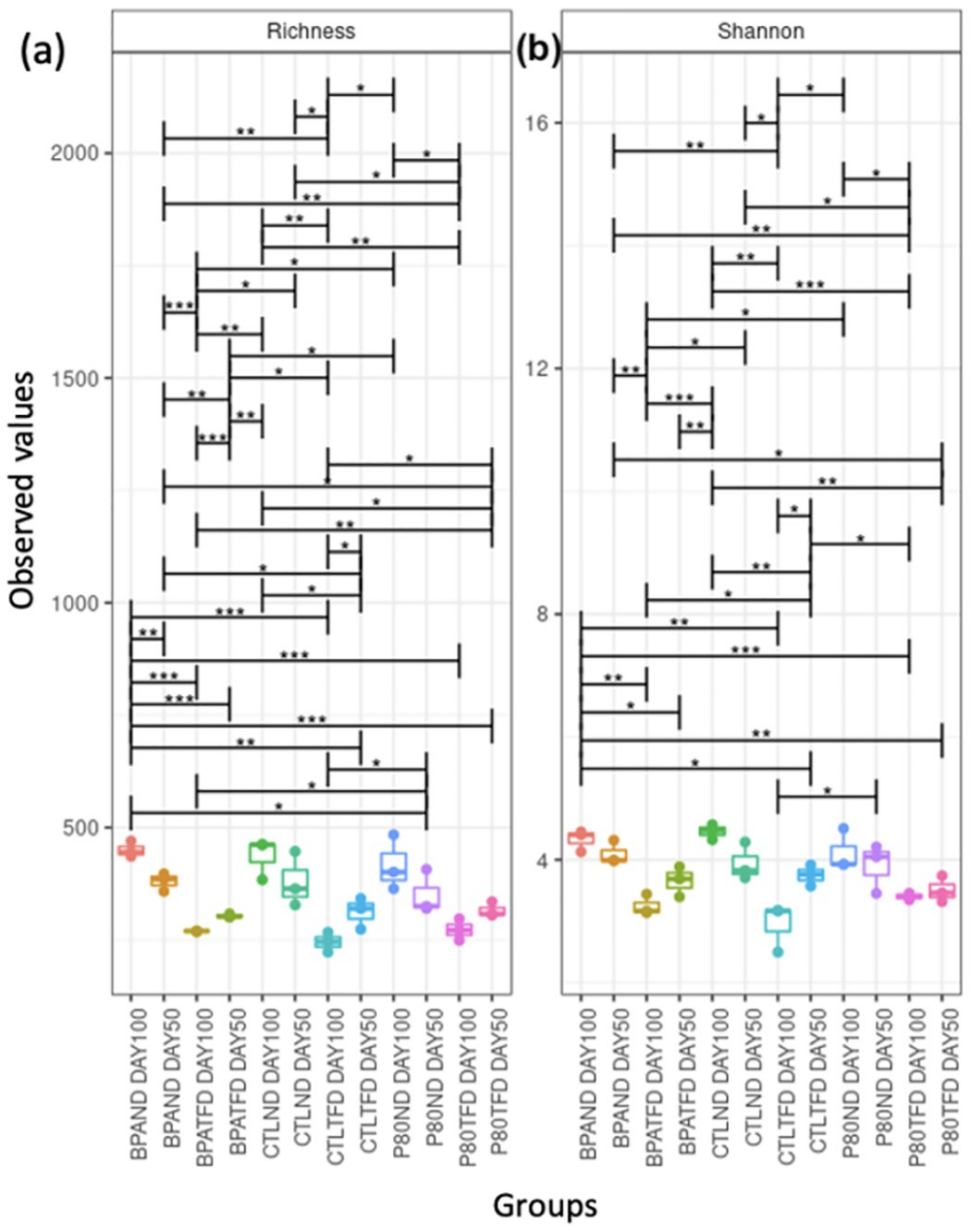
Alpha diversity of faecal microbiota collected from rat offspring at adolescence (day50) and adulthood (day100). (a) Bacterial species richness estimate (also known as Chao1) and (b) Shannon index. *p<0.05, **p<0.01, ***p<0.001; n = 3 per group.

The Chao1 species richness estimate and the Shannon index were used to investigate how prenatal BPA exposure and postnatal TFD influence the bacterial species richness and diversity within a sample (Fig 2a and 2b). Five main comparisons were made following the statistical significance reported in the alpha diversity parameter.

The first comparison made was to investigate the effect of postnatal TFD on rat offspring across all groups of prenatal exposure (control, vehicle, and BPA) on day 100. Chao1 was markedly reduced by 43.83% in CTLTFD (269.94 ± 27.15) compared to CTLND (480.58 ± 60.11; p<0.01) and 37.90% in P80TFD (299.47 ± 34.40) relative to P80ND (482.22 ± 75.96; p<0.05) (Fig 2a). Chao1 in BPATFD (300.72 ± 17.66) was also significantly lower than that of BPAND (498.11 ± 25.82; p<0.001) by 39.63% (Fig 2a). Likewise, Shannon indices were substantially decreased in CTLTFD (2.95 ± 0.23) relative to CTLND (4.46 ± 0.07; p<0.01) by 33.86% and by 17.48% in P80TFD (3.40 ± 0.03) compared to P80ND (4.12 ± 0.20; p<0.05) (Fig 2b). The same went with BPATFD’s Shannon index (3.25 ± 0.10; reduced by 24.94%) in comparison to that of BPAND (4.33 ± 0.10; p<0.01) (Fig 2b). In concordance with these findings, CTLTFD also demonstrated a significant decrease (by 45.81%) in bacterial species richness (269.94 ± 27.15; p<0.001) relative to BPAND (498.11 ± 25.82) (Fig 2a). Similarly, CTLTFD (2.95 ± 0.23; p<0.01) showed a significantly lower Shannon index than BPAND (4.33 ± 0.10) by 31.87% (Fig 2b).

The second comparison was to determine the alpha diversity of rat offspring’s intestinal flora at two different time points, which were adolescence (day 50) and adulthood (day 100). BPAND showed substantially lower Chao1 species richness estimated at adolescence (414.91 ± 22.23) compared to adulthood (498.11 ± 25.82; p<0.01) by 16.70%. Meanwhile, Chao1 of BPATFD was significantly higher (by 11.49%) on day 50 (339.75 ± 11.52) than on day 100 (300.72 ± 17.66; p<0.001) (Fig 2a). In addition, CTLTFD had substantially higher Chao1 (by 19.82%) at adolescence (336.68 ± 36.16) compared to in adulthood (269.94 ± 27.15; p<0.05) (Fig 2a).

Thirdly, rat offspring were compared to ascertain whether prenatal BPA treatment could affect the alpha diversity of their faecal microbiome. No significant difference in Shannon and Chao1 indices was observed when rat offspring were exposed to BPA prenatally (BPAND) compared to nonexposed rat offspring (CTLND and P80ND) (Fig 2a and 2b).

Next, the effect of the different types of control used, in other words, control water and vehicle water, on the intestinal flora alpha diversity was compared among rat offspring. Shannon and Chao 1 indices of rat offspring administered with vehicle water (P80ND) did not differ significantly relative to those administered with control water (CTLND) during the developmental period (Fig 2a and 2b).

Lastly, the synergistic effect of prenatal BPA and postnatal TFD on the alpha diversity of rat offspring’s gut microbiome was examined. There was no marked difference in the bacterial species richness and diversity in the combined treatment group (BPATFD) relative to BPAND rat offspring (Fig 2a and 2b).

**Fig 3:**
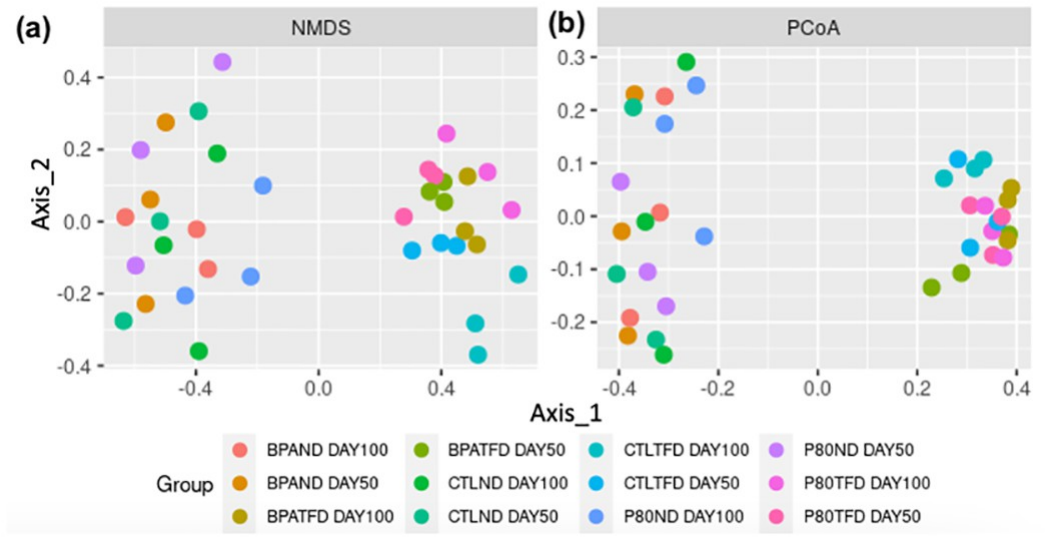
Beta diversity of faecal microbiota collected from rat offspring at adolescence (day50) and adulthood (day100). (a) Non-metric multidimensional scaling (NMDS) and (b) principal coordinates analysis (PCoA) plots, based on the unweighted and weighted UniFrac distance, respectively. n = 3 per group.

Meanwhile, beta diversity, which is used to analyse whether the bacterial communities differ between sample groups, was assessed by the NMDS and PCoA plots (Fig 3a and 3b). In this study, the former plot utilised the unweighted UniFrac distance (which is a quantitative measurement of beta diversity, for example, sequence abundance), while the latter employed the weighted UniFrac distance (which is a qualitative measurement of beta diversity, for instance, the presence and absence of sequences). The distance between samples (represented as colour-coded dots) indicated how varied the samples are in terms of their composition.

Rat offspring on TFD from all groups on days 50 and 100 were clustered together in the NMDS plot (Fig 3a). In contrast, rat offspring on ND were placed far apart from their TFD counterparts. A similar pattern was reflected in the PCoA plot (Fig 3b). Regardless, the microbial ecology of TFD samples was more closely related to each other in the PCoA plot than in the NMDS plot, while most ND samples consistently demonstrated that they are compositionally different from each other in both plots (Fig 3a and 3b).

### Effects of prenatal BPA exposure and postnatal TFD on the hierarchical cluster analysis of the adult rat offspring’s intestinal flora

**Fig 4:**
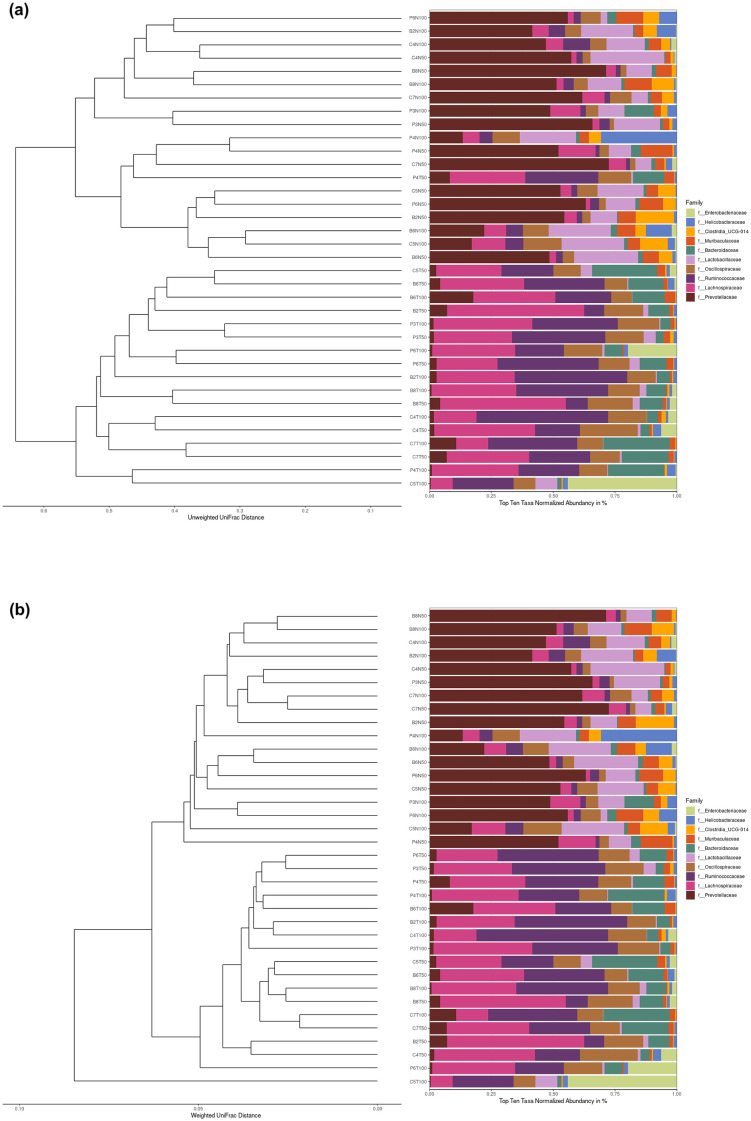
UPGMA cluster trees of the unweighted (a) and weighted (b) UniFrac distances at the family level. The clustering tree structure is on the left side, whereas the top ten relative abundance of different families for each sample is on the right side. Unweighted UniFrac included species evolutionary relationship and classification only, but is more sensitive to the low abundance features dissimilarities compared to weighted UniFrac. Species relative abundance was incorporated in the weighted UniFrac calculation, n = 3 per group.

Changes in the overall structure of the intestinal flora were also visualised using the hierarchical clustering method, specifically the unweighted pair group method with the arithmetic mean (UPGMA) tree, to detect trends in microbial community composition. Fig 4a and 4b show the respective UPGMA cluster trees of the unweighted and weighted UniFrac distances at the family level.

Unique cluster branches of the gut microbial community distinguished the ND and TFD groups, as depicted by the UPGMA tree of the unweighted UniFrac distance (Fig 4a). On the other hand, although the UPGMA tree of the weighted UniFrac distance exhibited similar groupings of the rat offspring (ND relative to TFD), one CTLTFD rat offspring on day 100 showed divergence from the other samples in the tree (Fig 4b). Moreover, the relative species abundance at the family level of the faecal sample was dominated by *Enterobacteriaceae*, while samples from the other TFD rat offspring were predominantly enriched by *Lachnospiraceae* and *Ruminococcaceae* (Fig 4a and 4b).

### Effects of prenatal BPA exposure and postnatal TFD on the microbial community profile of the adult rat offspring

**Fig 5:**
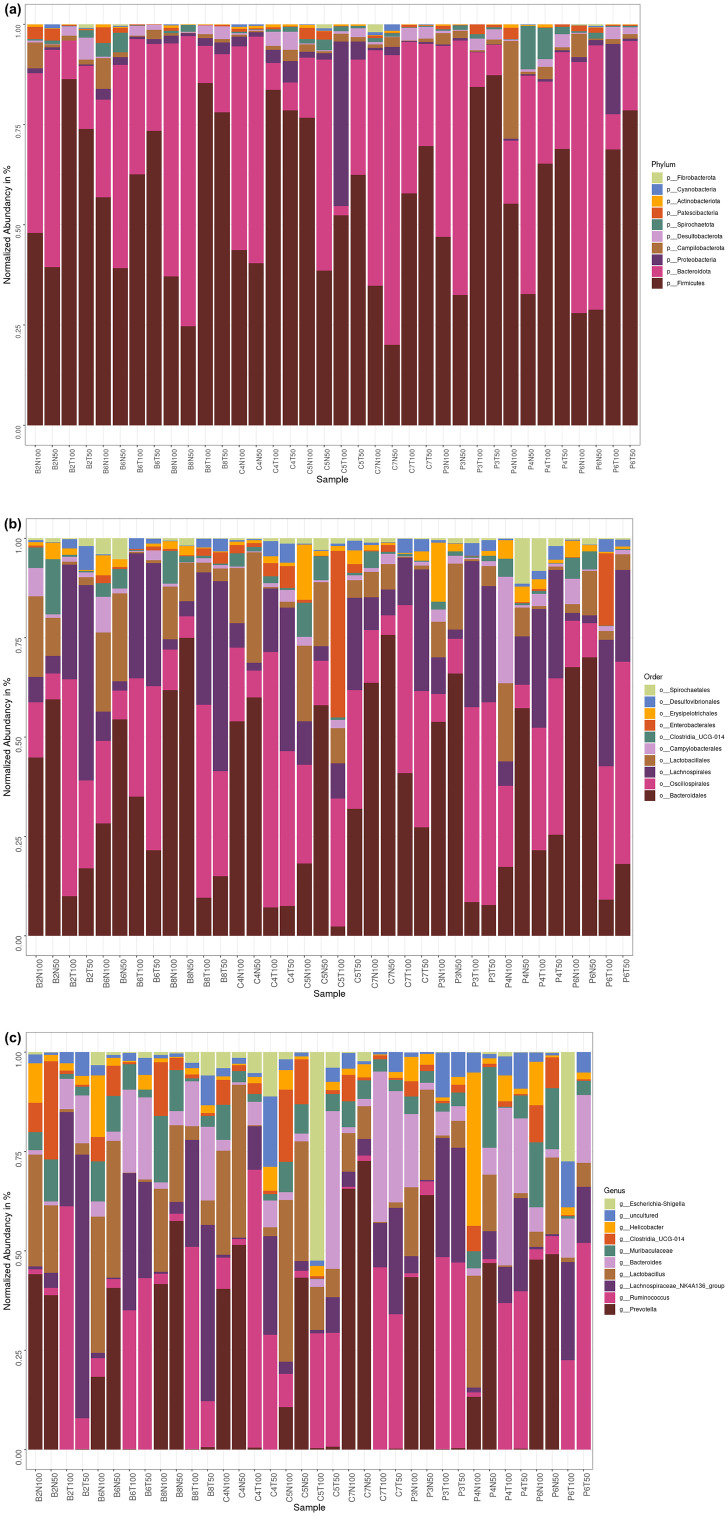
Profile of the offspring’s gut microbial community at different taxonomic levels. Bar plots showing the top ten most abundant bacterial phyla (a), (b) orders and genera (c) of the individual samples. n = 3 per group.

Fig 5a–5c shows the gut microbial community profile of the CTLND, CTLTFD, P80ND, P80TFD, BPAND and BPATFD offspring at different taxonomic levels, which are phylum, order and genus. The most abundant phylum in the TFD rat offspring was *Firmicutes*, followed by *Bacteroidota*, with one CTLTFD rat offspring on day 100 showing *Proteobacteria* as the second-most dominant phylum (Fig 5a). On the contrary, the order of the phyla *Firmicutes* and *Bacteroidota* distribution was reversed in the normal diet groups (CTLND, P80ND and BPAND), except for one CTLND and one P80ND rat offspring on day 100, which imitated the phyla distribution in the TFD rat offspring.

At the order level, *Bacteroidales* dominated the gut communities in the ND group (Fig 5b). In contrast, the intestinal flora in the TFD rat offspring was predominantly enriched by the order *Oscillospirales*, or *Lachnospirales* (mostly on day 50). However, one CTLTFD rat offspring on day 100 had *Enterobacterales* as the most abundant order.

Down to the genus level, *Prevotella* from the phylum *Bacteroidota* had the highest abundance in the ND group, while this species decreased substantially in the TFD rat offspring (Fig 5c). On the other hand, the predominant genera in the rat offspring on TFD were the *Lachnospiraceae NK4A136 group* and *Bacteroides* from the phyla *Firmicutes* and *Bacteroidota*, respectively. Both of these species were markedly reduced in the ND groups.

Overall, the trend exhibited by the taxa bar plots was in agreement with the alpha and beta diversity data of this study, wherein the gut microbiota composition of rat offspring was divided by diet, not by BPA treatment, nor the different stages of life or types of control used.

### Effects of prenatal BPA exposure and postnatal TFD on the network analyses of adult rat offspring’s intestinal flora

**Fig 6:**
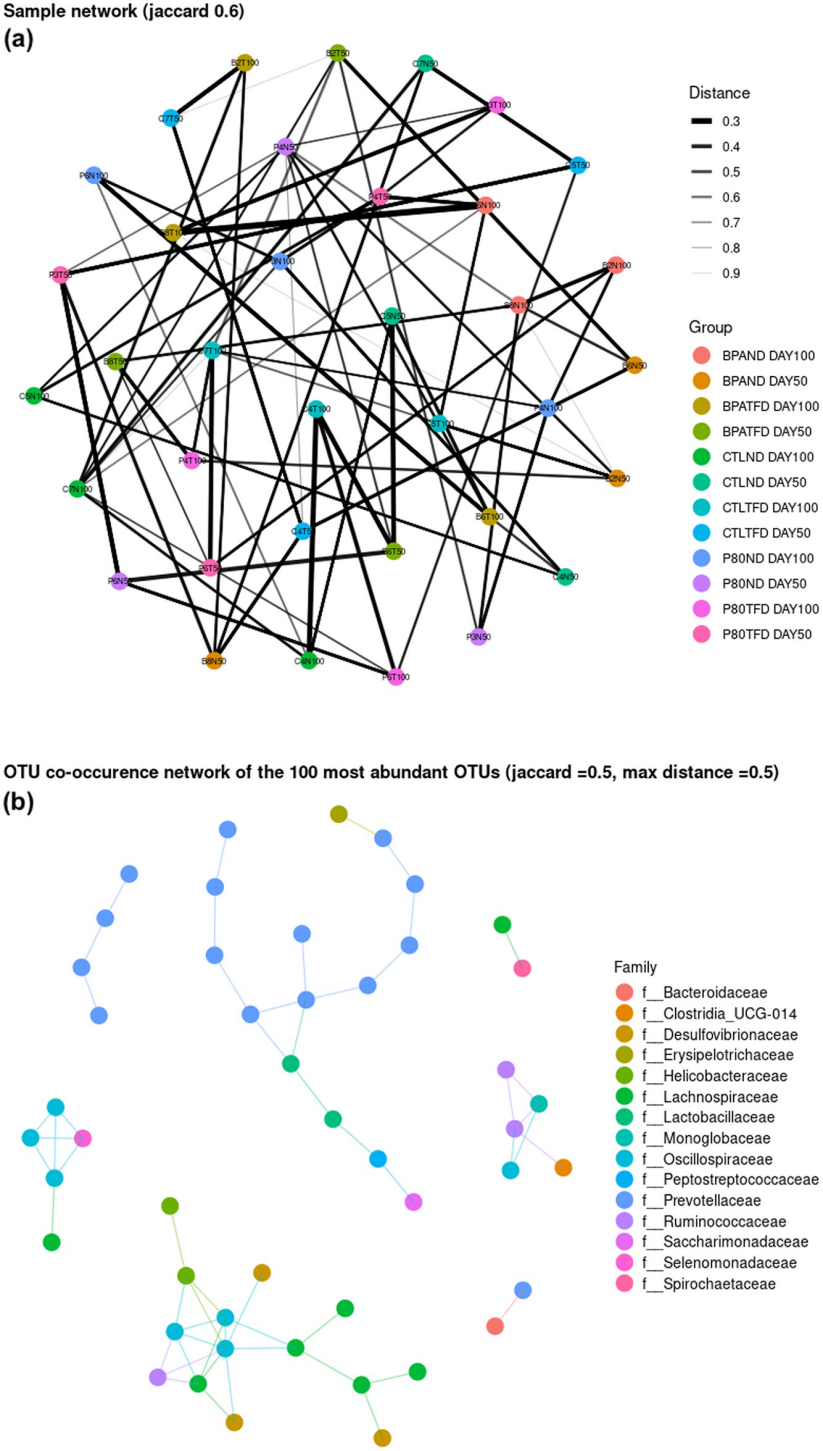
Network analyses of offspring faecal samples and bacterial families based on the jaccard correlation coefficients. (a) Sample network. The strength of co-relationship between samples was presented as the thickness of line, measured using the Jaccard distance. (b) Co-occurrence network of the 100 most abundant OTUs (presented as the colour-coded nodes) at the family level. Edges of the network represent the substantial interactions among nodes. n = 3 per group.

Network analyses of rat offspring’s faecal samples and their 100 most abundant OTUs at the family level were conducted to determine possible correlations between samples and the microbial taxa. This way, complex microbial community structures could be deciphered temporally or across the different experimental treatments. A Jaccard similarity index was used to build the networks to estimate the distances or extent of overlaps among samples (Fig 6a) and the bacterial families (Fig 6b). On the other hand, the dissimilarity between samples or microbial taxa is measured using the Jaccard distance, which complements the Jaccard coefficient.

There were 36 nodes (equal to all the faecal samples collected on day 50 and 100) and 80 edges (which signified how many connections formed between samples) identified in the sample network (Fig 6a). The Jaccard similarity index of all samples was 0.6, with the shortest distance between samples being 0.3 (for example, between BPATFD day 100 and BPAND day 100), and the longest being 0.9 (for instance, between BPATFD on day 50 and CTL TFD on day 50).

Meanwhile, the co-occurrence network of the 100 most abundant bacterial families exhibited a disassortative pattern with 47 nodes and 55 edges in total, with a Jaccard similarity index of 0.5 (Fig 6b).

A Jaccard similarity index close to 1.0 means that the samples or microbial taxa are more similar to each other, while an index close to 0 means that they barely share the same microbial community. Likewise, a shorter complementary Jaccard distance translates to a higher degree of dissimilarity between the samples or microbial taxa and vice versa.

### Effects of prenatal BPA exposure and postnatal TFD on the multivariate parametric analysis (DESeq2) of adult rat offspring’s intestinal flora

**Fig 7:**
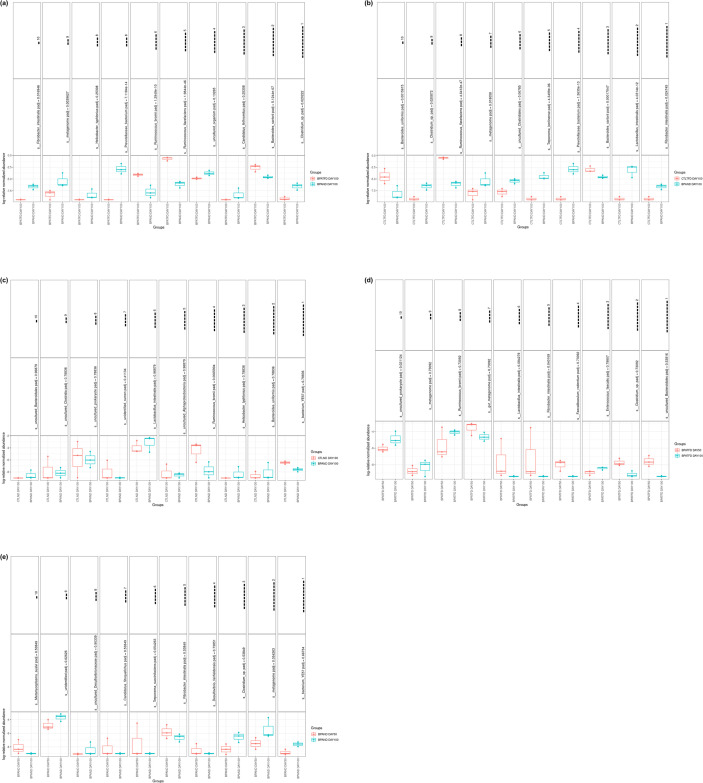
Multivariate parametric test using DESeq2. (a) BPATFD day 100 versus BPAND day100. (b) CTLTFD day 100 versus BPAND day 100. (c) CTLND day 100 versus BPAND day 100. (d) BPATFD day 50 versus BPATFD day 100. (e) BPAND day 50 versus BPAND day 100. n = 3 per group.

The microbiome of significantly differential abundance at the species level between two different rat offspring groups was identified using DESeq2 analysis. DESeq2 was chosen because it is more sensitive to small datasets (with less than 20 samples in each group) than large ones. Fig 7a–7e demonstrate the five comparisons made in this analysis, involving the stool samples from the CTLND, CTLTFD, BPAND, and BPATFD groups on days 50 and day 100.

The first comparison was between BPATFD and BPAND on day 100, which signified the effect of different diets on BPA exposed rat offspring (Fig 7a). For this pair, seven out of ten species were found to be significantly differentially abundant (p<0.0001–0.05). The species were: *Fibrobacter intestinalis*, *Metagenome*, *Prevotellaceae bacterium*, *Ruminococcus bromii*, *Ruminococcus flavefaciens*, *Bacteroides sartorii* and *Clostridium sp*.

The second comparison was between CTLTFD and BPAND on day 100, which implied whether the effect of prenatal BPA exposure imitated that of postnatal TFD in control rat offspring without Tween-80 (Fig 7b). There were nine substantially differentially abundant species observed when CTLTFD was compared to BPAND on day 100, such as *Bacteroides uniformis*, *Clostridium sp*., *Ruminococcus flavefaciens*, *Metagenome*, *Treponema berlinense*, *Prevotellaceae bacterium*, *Bacteroides sartorii*, *Lactobacillus intestinalis* and *Fibrobacter intestinalis* (p<0.0001–0.05).

The third comparison was between CTLND and BPAND on day 100, which indicated if there was a difference in response in the species differential abundance when exposed to BPA prenatally (Fig 7c). There was only one significantly differentially abundant species found, which was *Ruminococcus bromii* (p = 0.0065904).

Next, the comparison between BPATFD on day 50 and BPATFD on day 100 was to show how different ages affect species abundance in BPATFD rat offspring (Fig 7d). Uncultured prokaryotes and *Fibrobacter intestinalis* were the two substantially differentially abundant species observed in this pair (p<0.05).

Lastly, BPAND on day 100 was compared with BPAND on day 50 to demonstrate how species abundance changed at different life stages in BPAND rat offspring (Fig 7e). This pair did not show any significantly differentially abundant species.

Overall, the different postnatal diets may have higher significantly differentially abundant species than the different prenatal treatments and timepoints, which coincided with the alpha and beta diversity measures (Figs 2 and 3).

### Effects of prenatal BPA exposure and postnatal TFD on the metagenomic functional prediction of adult rat offspring’s intestinal flora

**Fig 8:**
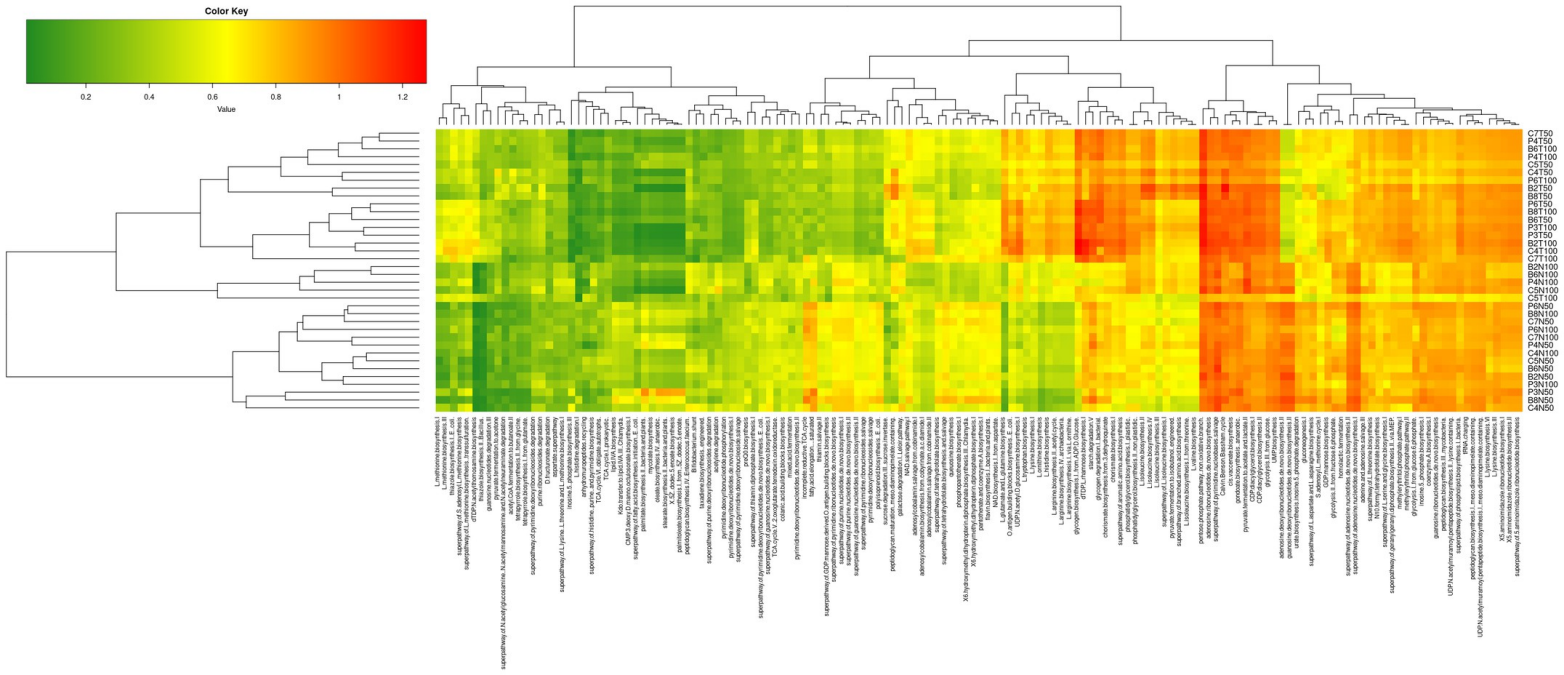
Bacterial metabolic functions predicted using PICRUSt2 and characterised based on KEGG orthologues. Each row is a sample, and each column refers to the KEGG level 4 pathway. Green indicates lower values, while red represents higher values. n = 3 per group.

An analysis of Phylogenetic Investigation of Communities by Reconstruction of Unobserved States 2 (PICRUSt2) was conducted to predict the functional profile of rat offspring’s metagenome. This analysis determined which KEGG (Kyoto Encyclopaedia of Genes and Genomes) pathway was affected by which factor. Fig 8 displays a heatmap divided into two parts: ND groups, filling the bottom half of the map, and TFD groups, occupying the upper half of the map.

**Fig 9:**
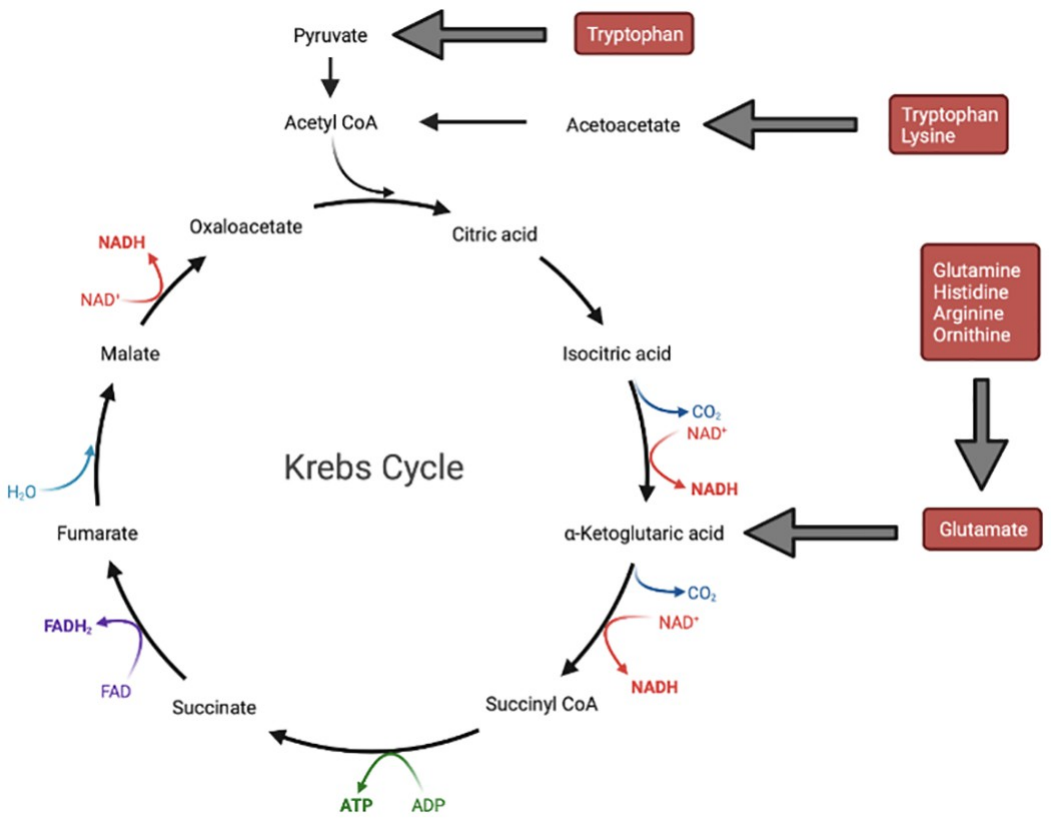
The perturbed amino acid metabolism pathway in TFD offspring based on PICRUSt2 functional prediction. Deamination of these amino acids will enable them to enter the glucose metabolism and tricarboxylic acid (Krebs) pathways [32]. Red boxes indicate the types of amino acids that may be perturbed, and grey arrows mean that there are intermediates in those reactions. This figure was created using Biorender.com.

The upregulation of pathways related to the biosynthesis of the amino acids (such as L glutamate, L glutamine, L tryptophan, L lysine I, L ornithine, L histidine, L arginine II (acetyl cycle), L arginine IV (archaebacteria), and L arginine I (via L ornithine)), was observed in TFD rat offspring relative to ND rat offspring (Figs 8 and 9). Likewise, pathways related to carbohydrate (for example, O antigen building blocks (E. coli) and UDP-N-acetyl-D-glucosamine) and glycan (for instance, glycogen biosynthesis I (from ADP-D-glucose)) biosynthesis were overexpressed in TFD rat offspring compared to ND rat offspring.

On the other hand, TFD groups displayed underexpression of the incomplete reductive TCA cycle-related pathway (which involves C1 compound utilisation and assimilation) and pathway related to FA elongation-saturated (which participate in FA and lipid biosynthesis) compared to ND groups (Fig 8). In addition, pathways related to nucleoside and nucleotide biosynthesis, such as adenosine deoxynucleotide de novo II and guanosine deoxynucleotide de novo II, were downregulated in TFD rat offspring in comparison to ND rat offspring.

## Discussion

The relationship between intestinal flora and obesity and microbial shifts post-BPA exposure have been well documented. Nonetheless, the interaction between prenatal BPA exposure and postnatal diet affecting the gut flora is yet to be explored, thus being evaluated in this study. Here, rat offspring were subjected to a daily dose of 5 mg/kg of BPA throughout the gestational period and were then placed on TFD for 11 weeks starting from day 21 after birth.

The alpha diversity analysis in this study revealed that there were no substantial differences between BPA-exposed (BPAND) and non-exposed offspring (CTLND) (Fig 2), which are in concordance with previous findings [33–35]. However, it is worth mentioning that prior experiments were conducted in a dissimilar manner, involving different biological species such as mice and rabbits and utilising varied levels of exposure ranging from 5 μg/kg/day to 200 g/kg/day [33–35]. Similarly, the alpha diversity measures were not greatly changed in mice on HFDs for 8 weeks relative to those fed with low-fat diets [36]. However, the Shannon index was markedly reduced in the high industrial TFA-content group, compared to the high control fat group [36]. Interestingly, comparable alpha and beta diversity indices were documented between BPA-exposed mice and mice fed with HFD and high-sucrose diet, showing that the intestinal flora structures of BPA-exposed mice emulated those of HFD-fed and high-sucrose-fed mice [20]. Meanwhile, the alpha diversity indices of BPAND in this study were significantly higher than those of CTLTFD rat offspring (Fig 2), indicating that BPA exposure effects do not resemble the TFD effects in adult rat offspring and that TFD has a stronger influence than BPA on rat offspring’s gut flora.

It is possible that the underlying mechanism to similar alpha diversity between BPA-exposed and non-exposed offspring is the fact that species richness and functional response diversity, which promote resilience, govern diversity [37]. Species richness is defined as the number of species present in a given system. According to ecological theory, species-rich communities use limited resources more efficiently and specialise to each resource, making them less susceptible to disturbances. Eutrophication, or excess nutrient loading, reduces ecosystem diversity and resilience because a few species outgrow and outcompete everything else. Consistent with this notion, decreased diversity has been linked with obesity and with a “Western” diet high in fat and sugar compared to those on low-fat plant-based diets, although whether this decreased microbiota diversity results in a decrease in resilience is unknown. Meanwhile, functional response diversity is defined as the degree to which species in a community that perform the same environmental function vary in their sensitivity to ecosystem changes. A rare but functionally similar species may fill a niche when an abundant species is damaged by an environmental perturbation due to high functional response diversity. For instance, a rare microbe may become abundant after antibiotic administration to fill an essential niche previously dominated by a microbe with higher antibiotic sensitivity, resulting in the persistence of the same stable state but decreased resilience due to functional redundancy. From another perspective, the nonmonotonic effect of BPA where higher dosage of BPA (mg/kg/day) has minimal impact on the measured effects may explain the no difference in alpha diversity observed in BPA-exposed and non-exposed offspring [38, 39].

This is supported by the beta diversity data, in which rat offspring were distinctly separated by postnatal diet but not by exposure during the developmental period (Fig 3). It is worth noting that unweighted UniFrac considers the presence and absence of sequences (qualitative), whereas weighted UniFrac uses sequence abundance (quantitative) [40]. In agreement, Ge et al. [36] showed distinct separation of the low-fat control, low-industrial TFA, high-fat control, and high-industrial TFA diets in the unweighted and weighted UniFrac PCoA plots. Furthermore, different studies exhibited that BPA-exposed and non-exposed offspring displayed no significant difference in their beta diversity values estimated using PERMANOVA of the weighted UniFrac distances [33, 35]. However, the beta diversity index of BPA rabbit offspring differed significantly from control rabbit offspring when calculated using the unweighted UniFrac distance [33]. Similar results were reported when different approaches were applied for the ordination, in which Bray-Curtis distance and Hellinger-transformed abundance were used [34]. Compared to UniFrac metrics, Bray-Curtis is not phylogenetically aware, where bacterial communities will appear equidistant in a PCoA plot, while UniFrac distance puts a less related bacterial community far from the more related ones [40]. Interestingly, faecal samples were also separated according to the age they were collected in this case, with the strongest outcome exhibited at week 20. Perhaps the effect of the different time points in this study was not striking enough due to the small sample size (n = 3 representative biological replicates from each group).

In line with the alpha and beta diversity data of the current study, the UPGMA trees of the weighted and unweighted UniFrac distances signified that the postnatal TFD significantly altered the structure of the gut microbiome in rat offspring relative to the exposure during in utero life (Fig 4). Moreover, the Jaccard similarity index of all the examined faecal samples was 0.6 (Fig 6a), which meant that about half of their microbial taxa were similar to each other. Although one CTLTFD rat offspring consistently displayed divergence from other samples in the UPGMA cluster tree of the weighted UniFrac distance and the relative abundance of the bacterial communities at the phylum, order and genus levels (Figs 4b, 5a–5c), the sample network demonstrated a high level of connectivity between rat offspring intestinal flora with an assortative pattern (Fig 6a). Meanwhile, the Jaccard similarity index of the bacterial taxa was 0.5 (Fig 6b), indicating that half of the bacterial families were similar. This explained why the maximum distance between OTUs was 0.5 (Fig 6b), which meant that half of the bacterial families were dissimilar to each other, which agreed with the UPGMA cluster tree of the unweighted UniFrac distance finding. Importantly, our results align with our previous findings, which showed that offspring on TFD were significantly heavier than their ND counterparts [41], which is consistent with previous research in obese humans and mice [36, 42, 43].

Besides, *Firmicutes* and *Bacteroidota* were the most abundant phyla in both CTL and TFD rat offspring (Fig 5a), which is in agreement with previous data on BPA-exposed rabbit offspring [33]. However, this finding contradicted the two dominant phyla of the industrial TFA groups from another study, which were *Firmicutes* and *Proteobacteria* [36]. In the current study, *Proteobacteria* was the second-most dominant phylum in one TFD sample only (CTLTFD on day 100), with *Enterobacteriaceae* as the dominant family (Fig 5a). Interestingly, it has been shown that type 2 diabetes mellitus (T2DM) and obese models exhibited a marked rise in the *Enterobacteriaceae* family and *Proteobacteria* phylum relative to the control groups [44]. Other than that, Ge et al. [36] showed an increase in *Desulfovibrionaceae* relative abundance (which is deemed as bad gut bacteria) and a decline in *Lachnospiraceae* and *Bacteroidetes* abundance (regarded as good gut bacteria) following industrial TFA intake. In the current study, the *Lachnospiraceae* family and *Bacteroides* genus were predominant in the TFD offspring compared to the ND offspring (Fig 5c). Multiple studies have demonstrated the role of both bacterial species in gastrointestinal diseases, such as inflammatory bowel disease and Crohn’s disease [45–48], with the former also involved in the glucose metabolism impairment and positively associated with leptin levels [49–52].

Furthermore, resembling the BPA impacts on colonic excreta of rabbit offspring from a previous study [33], higher *Ruminococcus* genus abundance, which belongs to the *Lachnospiraceae* and *Ruminococcacaea* families, was found in TFD rat offspring relative to CTL rat offspring, as demonstrated by DESeq2 and the top ten most abundant genera analyses (Figs 5c, 7a–7c). It has been documented that both of these families are key short-chain fatty acid (SCFA) producers, which maintain barrier integrity and have anti-inflammatory characteristics [53]. The gut bacterial metabolites, such as butyrate, have been shown to highly influence DNMT activity [54], by engaging in the one-carbon metabolism. In one study, the proliferation and differentiation of intestinal stem cells were shown to be regulated by the intestinal flora metabolites SCFAs [55], wherein isobutyrate levels, which were positively correlated with *Ruminococcacaea* abundance, assisted the intestinal stem cell proliferation via Wnt/beta-catenin pathway activation. Perhaps the increase in the *Ruminococcus* genus abundance could be a compensatory response to alleviate the intestinal injury caused by the TFD intake. In addition, *Lactobacillus intestinalis* which was significantly higher in BPAND rat offspring than CTLTFD rat offspring (Fig 7b), has been found to improve gut flora composition and gut barrier integrity in ovariectomised rats [56]. In another study, *Lactobacillus intestinalis* was reduced in the DSS-induced colitis mouse model [57].

Other than that, the functional prediction of the faecal microbiota (Fig 8) in this study showed that pathways related to amino acid metabolism were substantially increased in the TFD rat offspring relative to the ND rat offspring. This is in line with the previous finding where targeted analysis of BPA-exposed rabbit offspring’s global metabolite profiles exhibited an altered liver and colon amino acid metabolism resulting from gut bacterial dysbiosis [33]. In another study, the amino acid profile served as a biomarker in obese children, as reflected by the positive correlations between HOMA-IR and the different types of amino acids [58]. In addition, obese human and animal models have demonstrated an increase in branched-chain amino acid (BCAA) levels such as valine and isoleucine [59–61]. This increase led to the mammalian target of rapamycin complex 1 (mTORC1) activation and lipolysis inhibition in the skeletal muscle, further promoting obesity and insulin resistance development. Interestingly, it has been shown that hepatic inflammation and lipogenesis gene expressions were reduced by BCAA supplementation in rats fed with a HFD, which acted via elevated acetic acid synthesis and *Ruminococcus flavefaciens* abundance [61].

Furthermore, in one study, inverse associations were found between the metabolism of histidine, tryptophan, linoleic acid and arachidonic acid, with *Clostridiales* abundance (which was elevated in BPA mouse dams relative to their control counterparts) [25]. The same study also demonstrated positive associations between *Clostridium sp*. abundance (higher in control compared to BPA male mouse offspring) with glycolysis and starch and sucrose metabolism. Meanwhile, the current study exhibited that *Clostridium sp*. abundance and the biosynthesis of fatty acid (FA) and lipid were greatly decreased in TFD rat offspring compared to ND rat offspring, whereas carbohydrate (or glucose) and amino acid biosynthesis were increased (Figs 7a, 7b and 8). Likewise, T2DM patients exhibited a marked decrease in *Clostridium* levels relative to healthy controls [62], whereas O antigen building block biosynthesis was elevated in insulin-resistant obese children and adolescents compared to insulin-sensitive subjects [63]. On the other hand, it was previously reported that insulin-resistant individuals exhibited an increase in serum BCAA and N-acetyl-D-glucosamine, with decreased capacity for bacterial BCAA uptake; hence, less BCAA uptake by the microbial community and more are available for the host [64, 65].

In bacteria, glycogen synthesis is made from ADP-D-glucose [66], and this pathway was overexpressed in TFD rat offspring compared to ND rat offspring (Fig 8). A carbon capacitor (created when there are abundant carbon sources but lacking in other nutrients) regulating downstream carbon fluxes, the glycogen metabolic pathway supports persistent bacterial survival during unfavourable times [67, 68]. Moreover, associations between glycogen biosynthesis and degradation with energy storage compound synthesis and multiple key physiological functions, such as the metabolism of carbon and nitrogen, have been documented [67]. In this regard, *Lactobacilli* have been found to be involved in glycogen metabolic pathways [67], where it was elevated in CTLTFD rat offspring compared to BPAND rat offspring in this study (Fig 8), similar to those reported in some Crohn’s disease, obesity and T2DM cases [69–71]. Paradoxically, the FA elongation pathway producing saturated FAs was downregulated in the TFD groups relative to the ND groups (Fig 8). In one study, ablation of very long chain FA elongase 3 in mice attenuated their serum leptin and triglyceride levels and lipogenic gene expression in the liver, making them completely resistant to diet-induced obesity [72]. The diminished FA and lipid biosynthesis in the TFD groups (Fig 8) may be a compensatory response to the increase in amino acid and carbohydrate biosynthesis following the TFD intake.

Other than that, amino acids and their metabolites are involved in the tricarboxylic acid cycle (TCA; also known as the Krebs cycle), as shown in Fig 9. The purine nucleotide cycle has also been reported to be heavily involved in amino acid catabolism and flux regulation via the TCA cycle and glycolysis [73–75]. Importantly, it has been demonstrated that TCA intermediates, such as α-ketoglutaric acid, determine cell fate and function via epigenetic mechanisms, such as DNA methylation, apart from participating in macromolecule biosynthesis, for example, proteins, nucleotides and lipids [76, 77]. Moreover, TCA intermediates succinyl-coenzyme A (succinyl-CoA), oxaloacetate and 2-ketoglutarate could still be generated from pyruvate even if the bacteria harbour an incomplete TCA cycle [78]. However, this ability was reduced in TFD rat offspring relative to ND rat offspring (Fig 8). This could be the reason behind the increase in the upstream amino acid, carbohydrate, and glycogen biosynthesis pathways; thus, more of these metabolites are available for the host as there are fewer uptakes by the bacterial community. This could also explain the diminished downstream pathways of FA/lipid and nucleotide/nucleoside biosynthesis in the TFD groups compared to the ND groups (Fig 8). Unfortunately, the decrease in the nucleotide/nucleoside biosynthesis pathway in TFD offspring relative to ND offspring in the present study was not evident in their small intestinal global DNA methylation as reported in our previous study [79]. It is possible that dysregulated nucleotide/nucleoside biosynthesis occurred elsewhere, such as in the liver, where de novo purine nucleotide synthesis takes place. Furthermore, the dysregulation of the TCA cycle may conceivably result in discordant immune and stem cell function as well as tumorigenesis [77]. Ultimately, the metagenome functional prediction data of the current study mimicked those from a previous study investigating the salivary metabolic profile of obese paediatric patients with liver disease and metabolic syndrome, where the interaction between these pathways was illustrated as a metabolic systemic map [80].

The present study has several limitations. Specifically, we did not assess behavioural or cognitive repercussions in rat offspring. It is possible that an altered gut microbiome and metabolic disturbances could lead to the development of symptoms that resemble hyperactivity. This was observed by increased movement both before and after amphetamine injection in rats that consumed 10–20% TFA postnatally for 8 weeks [81]. A separate investigation found that consuming HFD for an extended period of 40 weeks led to an elevation of amyloid-β in the brain, which is indicative of cognitive decline [82]. Furthermore, the short period of observation in the current study makes it inconclusive whether altered gut microbiome in response to postnatal TFD influences susceptibility to metabolic diseases such as diabetes or obesity later in life. Okamura et al. discovered that consuming 40% TFAs for a period of 12 weeks led to an imbalance in gut bacteria (specifically, a greater amount of the *Desulfovibrionaceae* family, which belongs to the phylum Proteobacteria), resulting in immune alterations (such as increased CD36 expression) in the intestines of mice by week 20, significantly worsening metabolic disorders, such as diabetes and fatty liver, when compared to ND [22]. Moreover, the disruption of gut microbiota and metabolic disturbances discovered in this study may have long-lasting consequences for the rats as they age, potentially affecting their lifespan and geriatric health. It has been shown that gut dysbiosis at adolescence which can occur due to immature intestinal flora during early childhood may cause immune dysregulation and hormonal imbalance in adulthood, affecting health outcome such as neurodegenerative and atherosclerotic cardiovascular diseases [83]. On the other hand, in the presence of excessive nutrient intake and lack of physical activity, persistent activation of redox signalling pathways in individuals with metabolic syndrome may contribute to accelerated cellular ageing [84].

Not only that, research has also demonstrated that the formation of the gut microbiome and metabolic health in infants, which is influenced by the mother’s HFD-induced obesity as well as environmental exposures, may play a role in the transmission of obesity and metabolic disorders from one generation to the next [85, 86]. Although the impact of paternal gut dysbiosis and metabolic dysfunction on future generations remains unclear, this study would benefit from exploring the transgenerational effects of combined BPA exposure and TFD on the gut microbiome or metabolic health of subsequent generations. Previous research has found that when mothers (F0) were exposed to both BPA and HFD simultaneously, it led to elevated blood pressure in the second (F2) mouse generation [87]. This rise in blood pressure may be linked to lower levels of endothelial nitric oxide synthase (eNOS). In addition, the likelihood of obesity was higher in patrilineal female F2 compared to patrilineal male F2. In another study, exposure of F0 mothers to bisphenol S caused ileum and colon inflammation in F1 and F2 male offspring at adulthood [88]. Gastrointestinal inflammatory diseases are often linked to dysbiosis [89], therefore BPA exposure and TFD may affect future generations’ gut microbiome.

Another drawback of this study is the need to take into account the hormonal differences between male and female offspring, which could potentially impact the findings of the current investigation. Previous research has shown that mice subjected to a 16-week HFD (60% fat) and a 4-week high-protein diet exhibited disparities in the relative abundance of *Patescibacteria* [90, 91]. This phylum was found to be highly abundant in hepatocellular carcinoma patients and may be involved in non-alcoholic fatty liver disease pathogenesis. In our case, the same dietary intervention may have led to distinct changes in the gut flora of the female offspring, for example in *Patescibacteria* abundance. However, in another study, female mice were protected against diet-induced insulin resistance, whereas male mice were more susceptible to diet-induced weight gain [92].

Furthermore, this study only utilised two types of diets, ND and TFD. This study would benefit more with different types of HFDs, where prior research has indicated that HFDs (coconut oil (CO; saturated fats), a combination of conventional soybean oil (polyunsaturated fats) and CO, as well as a combination of genetically modified soybean oil (monounsaturated fats) and CO) lead to an increase in the populations of various harmful bacteria in both mouse small intestine and colon [93]. On the other hand, beneficial bacteria, such as segmented filamentous bacteria (SFB), Bacteroides, and *Prevotella oris*, are significantly reduced in the presence of HFDs. Wistar rats that were administered HFDs (42%) from lard and olive oil for 12 weeks had the highest levels of obesity and insulin resistance. However, rats fed HFDs from CO and fish oil showed insulin sensitivity levels that were comparable to those of the control group [94]. Meanwhile, male mouse offspring exposed to 10 and 50 μg/kg/day BPA experienced increased body weight, fasting glucose and insulin levels, as well as perigonadal and retroperitoneal fat [31, 95]. Consuming HFD (28.53–45% fat) worsened this increase, resulting in dyslipidaemia, obesity, glucose intolerance, and pancreatic β-cell damage between weeks 18 and 26. Nevertheless, to the best of our knowledge, the impact of HFDs on the gut microbiome of offspring exposed to BPA during gestation has not been studied yet. We hypothesise that the gut microbiome of these offspring could suffer from a similar exacerbation effect caused by HFDs depending on the age of observation. It has demonstrated that HFDs containing long-chain saturated fatty acids can alter the composition of the gut microbiota, leading to dysbiosis, inflammation, and an increased likelihood of developing obesity and metabolic syndrome [96].

Another improvement that can be made to the current study is the investigation the dose-response relationship between the level of trans fat consumption and the degree of gut microbiome alteration and metabolic disturbance in BPA-exposed offspring. Research has demonstrated a clear correlation between higher TFA consumption and elevated levels of low-density lipoprotein cholesterol [97]. Additionally, there is an augmented likelihood of developing cardiovascular disorders associated with increased TFA intake (Zhu et al., 2019). According to Food Standards Australia New Zealand, there is a correlation between TFA intake and reduction in high-density lipoprotein cholesterol levels [97]. However, the total impact was not considered substantial. A meta-analysis examining the relationship between TFA intake and the prevalence of T2DM found no effect on glucose metabolism when comparing diets with high levels of total TFA to diets with low levels of total TFA [98]. However, based on our understanding, no evidence has been identified regarding the dose-response association between the level of trans fat consumption and the degree of alteration in the gut flora. Ge et al. proposed that the gut microbiota responds actively to the quantity and type of dietary lipids, indicating that there may be a relationship between the amount of trans fat consumed and the extent of changes in the gut microbiome [36].

To the best of our knowledge, the molecular pathways by which TFD affects the gut flora of BPA-exposed offspring have yet to be explored. Nevertheless, research suggests that HFDs modulate metabolic and inflammatory signalling by affecting precursors such as bile acids, lipopolysaccharides (LPS), SCFAs, methylamines, and indoles [99, 100]. SCFAs regulate hunger, energy, glucose, and lipid metabolisms. Methylamines activate the NF-κB pathway, leading to endothelial dysfunction and inflammatory cytokine production. LPS causes inflammation via the NF-κB pathway, leading to increased inflammatory cytokine levels and obesity development. Meanwhile, secondary bile acids increase immune cell infiltration, insulin resistance, and lipid metabolism via farnesoid X receptors and Takeda G protein-coupled receptor 5, whereas indoles bind to intestinal barrier-maintaining pregnane X receptors. Future studies should consider these variables to investigate potential strategies for treating obesity and metabolic disturbances that result from gut dysbiosis.

The results of the present study suggest that the families *Lachnospiraceae* and *Ruminococcaceae* may serve as reliable predictors of metabolic disorders. Specifically, the former has been demonstrated to impede the process of glucose metabolism and exhibit a positive association with leptin levels [49–52]. Conversely, an increase in the quantity of *Ruminococcus flavefaciens* decreased liver inflammation and the expression of genes involved in fat production [61]. Future investigations could confirm the utility of *Lachnospiraceae* and *Ruminococcacaea* as biomarkers for predicting metabolic disturbances in both laboratory and clinical settings. To mitigate the negative effects of HFDs on gut dysbiosis and prevent an increase in body fat, a diet that incorporates unsaturated fatty acids and dietary supplements containing probiotics such as species from the *Lactobacillus* and *Bifidobacterium* genera, as well as prebiotics such as oligosaccharides, inulin, and polyphenols may be beneficial [101]. Additionally, resveratrol (a type of polyphenol) butyrate esters have been found to prevent obesity in female rats exposed to BPA during the perinatal period by reducing weight gain and lipid accumulation caused by BPA, optimising blood lipid levels, significantly decreasing the Firmicutes/Bacteroidetes ratio, and increasing the abundance of S24-7 while decreasing the abundance of *Lactobacillus* [102].

## Methods

### Ethics statement

All procedures involved in the experiment were carried out in compliance with the guidelines set by ARRIVE (Animal Research: Reporting of In Vivo Experiments) and were authorised by the Universiti Teknologi MARA (UiTM) Animal Research and Ethics Committee (approval number UiTM CARE: 294/2020 (7/2/2020)).

### Rats rearing and treatment

**Fig 10:**
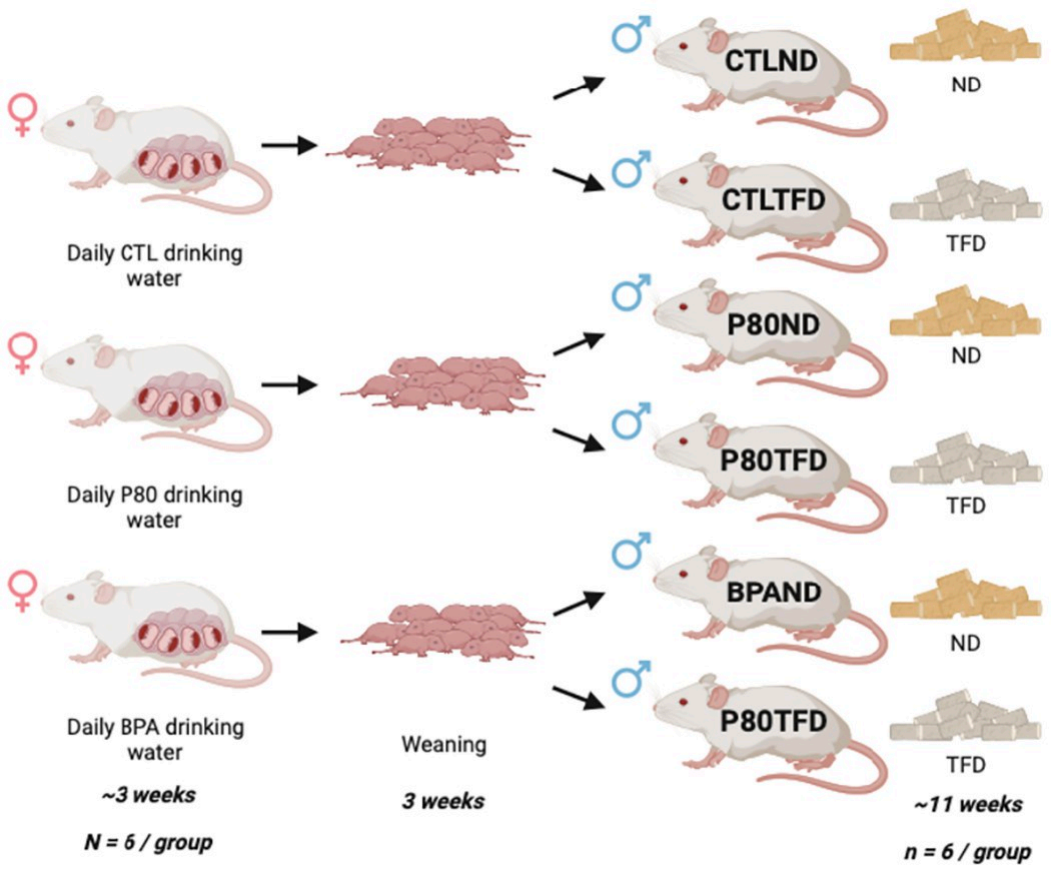
Graphical summary of the experimental procedure from the developmental period until early adulthood. One-third of the rat offspring were exposed to BPA prenatally (from pregnancy day 2 until birth). After weaning, male offspring from each pregnancy group were further divided into an ND or a TFD group. Faecal samples were collected on days 50 and 100 for microbial assessment. This figure was constructed using Biorender.com.

Rat offspring were prenatally exposed to either 5mg/kg/day BPA, a vehicle control consisting of Tween-80 (P80) or unspiked control (CTL) drinking water as described in our previous study [41]. Pregnant rats delivered the pups normally, and that day was marked as day 0. At day 21, male rat pups (determined by the distance between the anus and genital, which is twice as long as that of female rats) were separated from their dams and siblings and placed in individual cages in a temperature-controlled room (22°C) and 12-h light:12-h dark cycling, with access to food and water ad libitum until day 100. Male rat offspring were given either a normal diet (ND) or a trans fat diet (TFD; Research Diets, New Brunswick, NJ) via arbitrary selection using Microsoft Excel, as shown in Fig 10 (n = 6 rat offspring per group). The TFD provided 25 kcal% of its energy from fat, with the most abundant species being industrial TFA, trans-18:1, n-6 (elaidic acid) [103]. Female rat offspring were not included in the experiment because studies have shown that gut bacteria metabolism is modulated by oestrogen and progesterone levels; thus, they may affect the experimental outcome [104, 105]. On days 50 (puberty) and 100 (adulthood), faecal samples from rat offspring were collected by placing each rat offspring in metabolic cages to ensure that the stools were not contaminated with urine, bedding, or pellet crumbs. Stools were snap frozen before being stored in a -80°C freezer for gut microbiome analysis.

### DNA extraction

Thirty-six stool samples from the exposed and non-exposed rat offspring (three animals per group) were collected and immediately stored in the -80°C freezer until further analysed. A DNA isolation kit, the QIAamp PowerFecal DNA Kit (Qiagen, Germany) was used to extract DNA from 180 to 250 grams of the faecal samples, following the manufacturer’s instructions, with slight modifications. The extracted bacterial DNA was quantified using Quickdrop and used as a template to analyse the hypervariable V3-V4 region of the 16S bacterial rRNA. DNA integrity was checked on a 1% agarose gel with GelGreen stain. A 1% agarose gel was prepared by dissolving 1 gram of agarose in 100 ml of 1X Tris/borate/EDTA (TBE) buffer. One μl of loading dye was added to 5 ul of each sample prior to loading to well and electrophoresis was run at 80 kV voltage for 50 mins, together with 100 bp DNA ladder. The gel was visualised using the Bio-Rad Gel Doc XR+ Imager.

### V3-V4 16S rRNA gene region amplification and sequencing

Library preparation for 16S metagenomic sequencing and its analysis were outsourced and performed by Apical Scientific, Malaysia. DNA samples were subjected to three stages of quality control (quantification using Qubit, quality check on gel and PCR) prior to the construction of the 16S ribosomal RNA gene amplicons library in accordance with the Illumina manufacturer’s instructions. In general, two stages of PCR (amplicon and index PCR) were performed, followed by PCR clean-ups, before the library was quantified, normalised and pooled. Library denaturation was then performed before sample loading on the Illumina MiSeq platform. The V3 to V4 hypervariable region was amplified using specific primer sets in the presence of overhang adapters. Briefly, the amplification conditions were performed on a thermocycler by using the following conditions: 95 °C for 3 mins, 25 cycles of (95 °C for 30 secs, 55 °C for 30 secs, and 72 °C for 30 secs), and a final extension at 72 °C for 5 min. 16S V3 and V4 amplicons were purified from free primers and primer dimer species using AMPure XP beads. During the index PCR, the Nextera XT Index Kit was used to attach Illumina sequencing adapters and dual indices to the amplicons, using similar conditions but only 8 cycles for the second step of PCR. The fluorometric method was used to quantify libraries then libraries were diluted to a concentration of 4 nM for normalisation before pooling. Lastly, denaturation of the library pool (into single strand DNA) with sodium hydroxide followed by heat was done for cluster generation and sequencing, including an internal control (5% PhiX) which must be included in each run. The raw Illumina data produced in this study can be accessed from the NCBI Sequence Read Archive database using the accession number PRJNA1005630.

### Amplicon bioinformatics analysis

Before proceeding with statistical analysis of the microbiome data, the raw amplicon sequence data underwent a quality control (QC) process first prior to Operational Taxonomic Units (OTUs) clustering and taxonomic assignment. BBDuk from the BBTools package was used to remove the sequence adaptors and low-quality reads from the paired-end reads. Afterwards, merging the forward and reverse reads using USEARCH version 11.0.667 was performed to ensure that sequences exceeding 600bp and shorter than 150bp were excluded from the downstream processing. Next, alignment of reads with internal transcribed spacers (UNITE database) or 16S rRNA (SILVA database release 132) was performed, followed by chimeric error inspection using VSEARCH version 2.6.2. UPARSE version 11.0.667 was then utilised to cluster reads de novo into OTUs at 97% similarity, with rare OTUs (less than two reads or doubleton) being filtered out due to the spurious nature of MiSeq chemistry. After that, a phylogenetic tree was constructed by aligning a single representative sequence from each OTU (which was arbitrarily picked) against the SILVA 16S rRNA database using Pynast. Lastly, Quantitative Insights Into Microbial Ecology (QIIME) version 1.9.1 was used to assign taxonomies to OTUs against the SILVA 16S rRNA database.

Bioinformatics data were statistically analysed using several packages from R (version 3.6.1) and visualised using ggplot2. A pairwise ANOVA was performed using the R package vegan to evaluate alpha diversity (Chao1 species richness and Shannon index) between groups, in which the threshold for significance was set at p<0.05. The same package was also used to analyse beta diversity (non-metric multidimensional scaling, NMDS and principal coordinates analysis, PCoA plots), which employed the unweighted and weighted UniFrac distance matrices. Meanwhile, phylogenetic analysis (hierarchical clustering) was performed using the Phyloseq package, whereas the Microbiomeutilities package was used to check for sequencing depth. Lastly, metagenomic functions of Kyoto Encyclopaedia of Genes and Genomes (KEGG) pathways were predicted using the Tax4Fun2 package, which utilised the SILVA database.

## Conclusion

The intestinal flora composition in rat offspring appears to be shaped by postnatal diet but not by early life exposure to BPA. Our findings are suggestive of disrupted metabolic functions and epigenetic changes, particularly DNA methylation, in offspring consuming TFD, which could potentially lead to obesity manifestations in middle or late adulthood. Further research is needed to validate these interesting findings, including the impact of the combined BPA exposure and TFD on the gut microbiome and metabolic health of subsequent offspring generations.
